# Postprandial metabolic response of breast-fed infants and infants fed lactose-free vs regular infant formula: A randomized controlled trial

**DOI:** 10.1038/s41598-017-03975-4

**Published:** 2017-06-16

**Authors:** Carolyn M. Slupsky, Xuan He, Olle Hernell, Yvonne Andersson, Colin Rudolph, Bo Lönnerdal, Christina E. West

**Affiliations:** 1 Department of Nutrition, University of California Davis, One Shields Ave, Davis, CA 95616 USA; 2 Department of Clinical Sciences, Pediatrics, SE 901 85 Umeå University, Umea, Sweden; 3 Mead Johnson Nutrition, 2400 W Lloyd Expy, Evansville, Indiana, 47712 USA; 4 University of California, San Francisco, San Francisco, 94143 USA

## Abstract

Lactose intolerance is a major concern driving the growth of lactose-free foods including lactose-free infant formula. It is unknown what the metabolic consequence is of consumption of a formula where lactose has been replaced with corn syrup solids (CSS). Here, a randomized double-blinded intervention study was conducted where exclusively formula-fed infants were fed formula containing either lactose or CSS-based infant formula and compared with an equal number of exclusively breast-fed infants. Plasma metabolites and insulin were measured at baseline, 15, 30, 60, 90 and 120 min after feeding. Differences in plasma metabolite profiles for formula-fed infants included a rapid increase in circulating amino acids, creatinine and urea compared with breast-fed infants. At 120 min post-feeding, insulin was significantly elevated in formula-fed compared with breast-fed infants. Infants fed lactose-based formula had the highest levels of glucose at 120 min, and leucine, isoleucine, valine and proline at 90 and 120 min, whereas infants fed CSS-based formula had the lowest levels of non-esterified fatty acids at all time points, and glucose at 120 min. Overall, these differences highlight that changes in infant formula composition impact infant metabolism, and show that metabolomics is a powerful tool to help with development of improved infant formulas.

## Introduction

Lactose is the major carbohydrate in human milk, followed by oligosaccharides and glucose^1^. Compared with other mammals, human milk has the highest lactose concentration, 50% higher than cow’s milk^2^, contributing to over 60% of the total osmolarity in human milk^3^. We have previously reported that although the concentration of lactose increases gradually over time, individual variability is low compared with other milk sugars^1, 4^. In order to match the composition of human milk, milk-based infant formulas usually use lactose as the only, or main carbohydrate source. However, in recent years, lactose-free formulas containing maltodextrin (MDX) or corn syrup solids (CSS) have become increasingly used. Indeed, the National Health and Nutrition Examination Survey (NHANES) reported approximately 5% of infants received lactose-reduced formulas in the U.S alone between 2003 and 2010^5^, and this trend is increasing. A common rationale for the use of lactose-free infant formulas is that infants are presumed to be lactose intolerant; although there is little or no evidence that lactose reduced formulas are beneficial^5–7^.

We, and others, have previously shown that early diet is important in shaping the immune function, gut microbiota and metabolism of neonates^8–14^. However, none of these studies have examined the metabolic postprandial response of full-term healthy infants who were breast-fed or formula-fed, and the metabolic consequences of replacing lactose with another carbohydrate. Here, we present a randomized double-blind intervention study comparing the pre- and post-prandial response of serum insulin and plasma metabolites in 3-months-old infants upon consumption of human milk, lactose- or CSS-based formula.

## Results

Postprandial venous blood from 30 term infants (males:females, 50:50, with equal numbers of each sex in each group) with a mean age of 85.8 ± 4.8 days was obtained. No significant differences among the groups concerning mother’s age at delivery, parent’s education level, parity, gestational age as well as birth and test day length, weight and head circumference were observed (one-way ANOVA followed by post-hoc Tukey HSD test). Participants’ characteristics are presented in Supplementary Table 1. The average test meal consumed and feeding time was not significantly different between groups (one-way ANOVA followed by post-hoc Tukey HSD test).

### Pre-existing metabolic disparity between breast-fed and formula-fed infants at baseline

**Figure 1:**
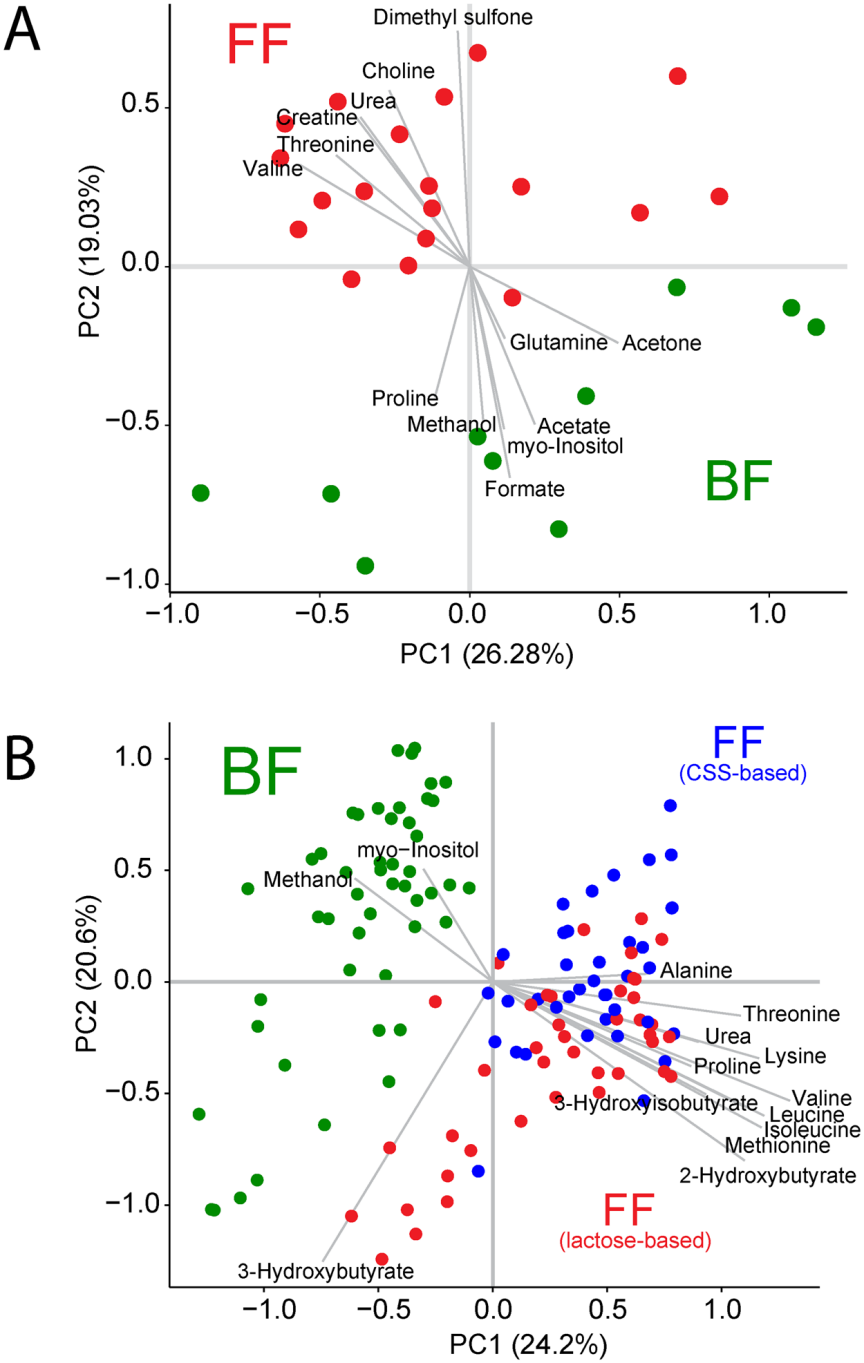
Principal components analysis (PCA) reveals differences between breastfed and formula-fed infants at baseline and after feeding. (**A**) PCA of baseline plasma samples from breastfed infants (green) and formula-fed infants (red). All formula fed infants were grouped together since all were consuming regular infant formula prior to the start of the experiment. Infants were acclimated to the taste of the formula for 1–3 feedings prior to the experiment. (**B**) PCA of plasma post-prandial metabolites from infants that were breastfed (green), infants that were fed lactose-based infant formula (red), and infants that were fed corn-syrup solids (CSS)-based infant formula (blue).

There were no significant differences between breast-fed and formula-fed infants at baseline for glucose, insulin or NEFA concentrations (one-way ANOVA followed by post-hoc Tukey HSD test). However, using cluster-based analysis, differences in the overall plasma metabolic profile at baseline were observed (Fig. 1A), reflecting a pre-existing metabolic difference between breast-fed and formula-fed infants prior to the start of the experiment. The average effect size (Cohen d) of all measured metabolites (35 total) at baseline (in the semi-fasted state) was moderate (0.79 and 0.77) between breastfed and infants fed CSS- or lactose-based formula respectively. Breast-fed infants had significantly higher acetate, acetone, formate, glutamine, methanol, proline and myo-inositol. In contrast, formula-fed infants had higher urea, creatine, essential amino acids (threonine and valine) and their amino acid catabolism by-products (2-hydroxybutyrate and 3-hydroxyisobutyrate), choline, and the host-microbial co-metabolite dimethyl sulfone^15^ (Table 1). There were no significant differences in the overall composition or individual metabolite concentrations between formula-fed infants assigned to the lactose- or CSS-based formula at baseline (one-way ANOVA followed by post-hoc Tukey HSD test, data not shown), although a weak average effect size of 0.45 for all measured metabolites at baseline (semi-fasted state) between infants fed CSS and infants fed lactose-based formula was observed.

### Acute response to diet indicates a postprandial metabolic modification by feeding

**Figure 2:**
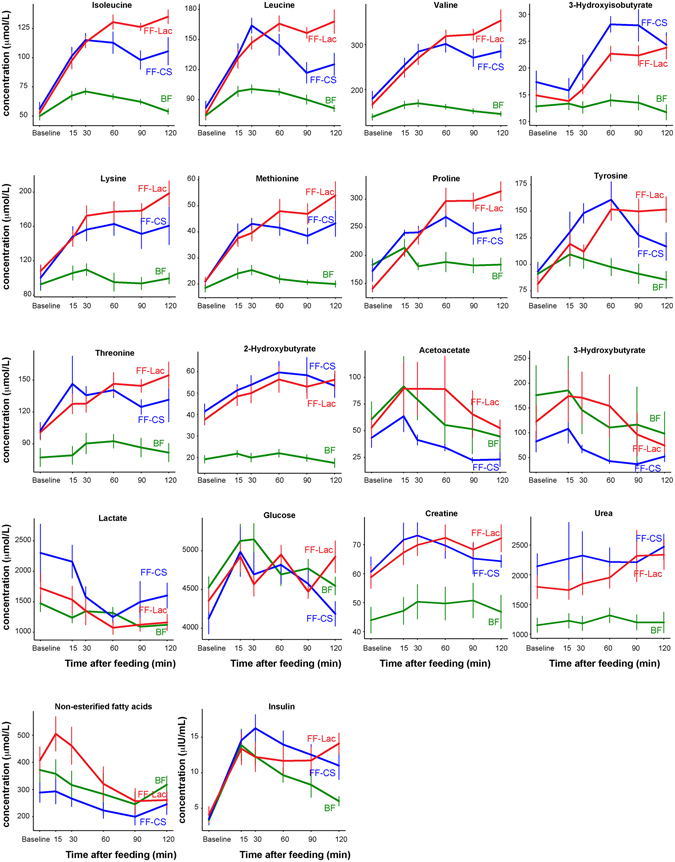
Post-prandial concentration of plasma metabolites and serum insulin. In green are concentrations in the plasma or serum of breastfed infants; red are infants fed lactose-based formula; and in blue are infants fed CSS-based formula.

Analysis of the postprandial plasma metabolome revealed a clear difference between breast-fed and formula-fed infants (Fig. 1B), with some separation between the infants fed lactose-based formula, and those fed CSS-based formula. The average post-prandial effect size for all metabolites was strong (1.57 and 1.65) between breastfed and infants fed CSS- or lactose-based formula respectively, and moderate (0.71) between infants fed CSS and infants fed lactose-based formula. The average effect size was strongest between the formula groups at 90 and 120 min (0.83 at each time point). Applications of ANCOVA, with post-meal measurements as outcome, and baseline measurements as covariate to eliminate baseline bias, suggest that except for proline (which was higher in breast-fed infants at baseline and increased significantly postprandially for formula-fed infants), all metabolites that were significantly different at baseline followed the same trend in the postprandial period (Supplementary Table 1). Compared with breast-fed infants, formula-fed infants had higher concentrations of circulating amino acids including alanine, the branched chain amino acids (BCAA: isoleucine, leucine, valine), lysine, methionine, proline, and tyrosine (Fig. 2).

**Figure 3:**
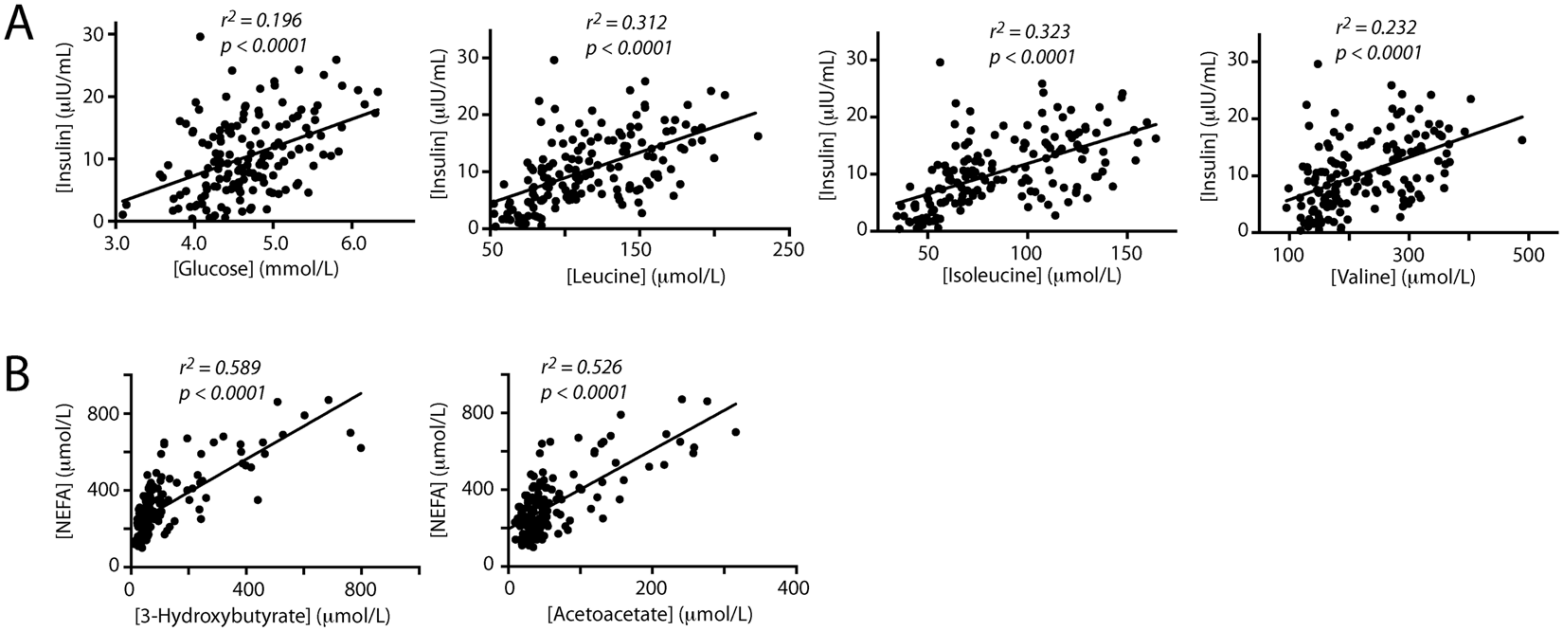
Correlation of serum metabolite concentrations. (**A**) Correlation of serum insulin concentrations (in μIU/mL) with plasma glucose, isoleucine, leucine or valine (in μmoles/L). (**B**) Correlation of serum NEFA (in μmoles/L) with plasma acetoacetate or 3-hydroxybutyrate (in μmoles/L). The correlation coefficient and p-value are indicated for each.

In the postprandial state, both plasma glucose and serum insulin levels were elevated compared with baseline, peaking at 15 or 30 min post-meal, as were BCAA (Fig. 2). Insulin and glucose were positively correlated, as was insulin with each of the BCAA (Fig. 3A). Although no overall difference in insulin and glucose concentrations based on AUC analysis (with or without adjustment of baseline; one-way ANOVA followed by post-hoc Tukey HSD test) was observed, at 120 min post-meal, both formula-fed groups showed elevated insulin compared with breast-fed infants (Fig. 2, and Supplementary Fig. 1A). Strikingly, those infants fed lactose-based infant formula had the highest average insulin compared to breast-fed infants, and significantly higher glucose compared with CSS-based formula-fed infants, whereas CSS-based formula-fed infants had the highest lactate at 15 min and lowest glucose at 120 min (Fig. 2, and Supplementary Fig. 1A). At both 90 and 120 min post-meal, plasma proline and BCAA were significantly higher in infants fed lactose-based formula compared with those fed CSS-based formula (Fig. 2, and Supplementary Fig. 2).

Ketone bodies (3-hydroxybutyrate and acetoacetate) were significantly higher in the lactose-based, compared with the CSS-based formula-fed infants (rmANCOVA, Fig. 2), and were similar to breast-fed infants. Concomitant with this, NEFA was elevated at 15 and 30 min post-meal in the lactose-based formula-fed infants compared to the CSS-based formula-fed infants (one-way ANOVA followed by posthoc Tukey HSD test, p = 0.06 and p = 0.03 respectively, Fig. 2, and Supplementary Fig. 1B), and was positively correlated with concentrations of plasma 3-hydroxybutyrate and acetoacetate (Fig. 3B).

## Discussion

Several studies have assessed metabolic differences between breast-fed and formula-fed infants^8–14^ (reviewed in refs 16, 17). However, none of these looked at the post-prandial metabolome in response to feeding. Moreover, no studies to date detailing the effect of consumption of a carbohydrate other than lactose on infant metabolism have been reported.

A profound metabolic discrepancy between breast-fed and formula-fed infants at baseline (semi-fasting state) suggests that overall infant metabolism is shaped by diet. Compared to formula-fed infants, breast-fed infants showed increased β-oxidation reflected as higher acetone. Formula-fed infants showed elevated protein degradation pathways with elevated nitrogen waste products (urea), creatine, essential amino acids (threonine and valine), and amino acid catabolism by-products (2-hydroxybutyrate, 3-hydroxyisobutyrate) consistent with their higher overall protein intake. Interestingly, the plasma concentration of 2-hydroxybutyrate, a product of threonine metabolism, was double in the formula-fed compared with breast-fed infants at baseline, and nearly triple 1 h into the post-prandial period. It was recently shown that 2-hydroxybutyrate may be an early marker for insulin resistance and impaired glucose regulation^18^; however, whether this holds true in infants remains to be shown.

Peroxisome proliferator-activated receptor-α (PPARα) has been identified as the master regulator of hepatic lipid metabolism (including fatty acid β-oxidation and ketogenesis), and additionally plays a role in glucose, amino acid, and lipoprotein metabolism^19^. For example, PPARα-null mice show increased circulating amino acids and urea^20^. In the semi-fasted state, we observed higher circulating threonine and valine (and their catabolic products 2-hydroxybutyrate and 3-hydroxyisobutyrate respectively) and urea in both formula groups compared with breast-fed infants, and lower acetone. Reduced PPARα activity in formula-fed infants compared with breast-fed infants could be one mechanism supporting these observations.

After feeding, a rapid elevation of circulating amino acids was observed in all infants as early as 15 min post-feeding (Fig. 2). It has previously been shown that there is a linear correlation between dietary amino acid intake and plasma amino acid levels^21^. Infant formula contains higher protein levels than human milk^22^, and in this study contained approximately twice that of human milk. Interestingly, the rise in plasma amino acid levels of the formula-fed infants was twice that of breast-fed infants at 30 min. Moreover, for breast-fed infants, circulating amino acids started to decrease after 30 min, whereas for infants fed CSS-based formula, this did not occur until after 60 min. However, for infants fed lactose-based formula, circulating amino acids continued to increase until 60 min, and remained high until 120 min (Fig. 2). This post-prandial difference may be due to differences in gastric emptying times. Breast milk has been shown to empty faster than lactose-containing formula, with the mean half-emptying times measured as 48 versus 78 min respectively^23^. Moreover, in premature infants, use of a glucose polymer (polycose) instead of lactose resulted in significantly less volume of gastric contents at 60 and 80 min^24^. It is therefore plausible that gastric emptying time is responsible for the differences in plasma amino acid concentration at the later time points in the post-prandial period between infants fed CSS- or lactose-based formula.

There is mounting evidence that suggests that leucine (as well as the other BCAAs) regulate mTORC1-S6K1 signaling, which may lead to insulin resistance^25^. Furthermore, mTORC1 controls adipogenesis through activation of PPARγ^26^. We observed an average 108% increase in plasma leucine over the first 30 min in both formula groups whereas leucine in breast-fed infants only increased by an average of 42%. Throughout the postprandial period, leucine remained high for the formula-fed infants (on average 87% higher and 148% higher than baseline in CSS- and lactose-based formula-fed infants respectively), whereas its concentration returned to near baseline values for breast-fed infants by 120 min. Exaggerated mTORC1 signaling during postnatal growth may promote mTORC1/SREBP-1/ PPARγ-mediated adiposity^25^. Indeed, formula-feeding has been shown to result in higher fat mass than breast-feeding^27^; however, no evidence of an increased risk of type 2 diabetes has been reported^28–30^.

Other postprandial differences in the plasma metabolome included increased urea concentrations, as well as lower *myo*-inositol, formate, methanol, and acetone in formula-fed relative to breast-fed infants. A similar finding was reported in our previous work investigating the effect of feeding practice on infant rhesus monkeys from birth to 3 months of age^13^. These results may be interpreted as formula-fed infants tending to favor the use of protein (higher protein:energy ratio), and breast-fed infants favoring use of fat for energy.

Lactose is a disaccharide composed of glucose and galactose. After digestion in the small intestine by lactase, more than 90% of the absorbed galactose is consumed by the liver^31^, where it is converted to glucose 1-phosphate that can subsequently be used for glycogenesis^31^. It was previously shown that the perfused neonatal rhesus liver is able to rapidly take up galactose when a combination of galactose, insulin and glucose are available, increasing glycogen synthase activity and inactivating phosphorylase^32^. Interestingly, no net glycogen synthesis was observed in response to glucose perfusion alone^32^. The increase in glycogen synthase activity achieved by consumption of lactose-based formula (as opposed to formula containing glucose polymers) suggests that more of the energy from the carbohydrate is converted to glycogen rather than directly to fat.

Little difference between infants consuming CSS-based and lactose-based formula was observed at baseline, likely due to the short lead-in period of 1–3 feedings prior to the experiment. However, while lower levels of post-prandial plasma amino acids could be explained by a difference in gastric emptying time, infants consuming the CSS-based formula had higher lactate at 15 and 30 min, and the lowest NEFA at all time points. Additionally, at 120 min, CSS-based formula-fed infants had significantly higher insulin than breast-fed infants, but the lowest average glucose compared with infants fed the lactose-based formula or breast-fed infants. It is known that high circulating NEFA activates ketogenesis^33^. Indeed, a strong correlation between circulating NEFA and 3-hydroxybutyrate and acetoacetate concentrations was observed (Fig. 3B). Low ketones and NEFA in the CSS-based formula-fed infants may have a negative impact on infant development, as although glucose is the main carbohydrate fuel for brain growth and metabolism^34, 35^, it is known that ketones are used to maintain normoglycemia in the infant, protecting the brain in case of hypoglycemia^16^. The mechanism behind the difference in circulating NEFA and ketones between infants fed CSS-based versus lactose-based formula is unknown at this time. Possibly, galactose increases lipolysis, as has been previously shown in obese women^36^.

In conclusion, this is the first study to show semi-fasted and postprandial differences in the plasma metabolome between breast-fed and formula-fed infants, and the effects of replacing lactose with CSS. Study limitations include a small sample size and lack of a longer follow-up period. Nonetheless, this study illustrates the power of metabolomics to uncover the effects of feeding on metabolism and shows how this technique is invaluable for development of improved infant formulas.

## Methods

### Study Design

**Figure 4:**
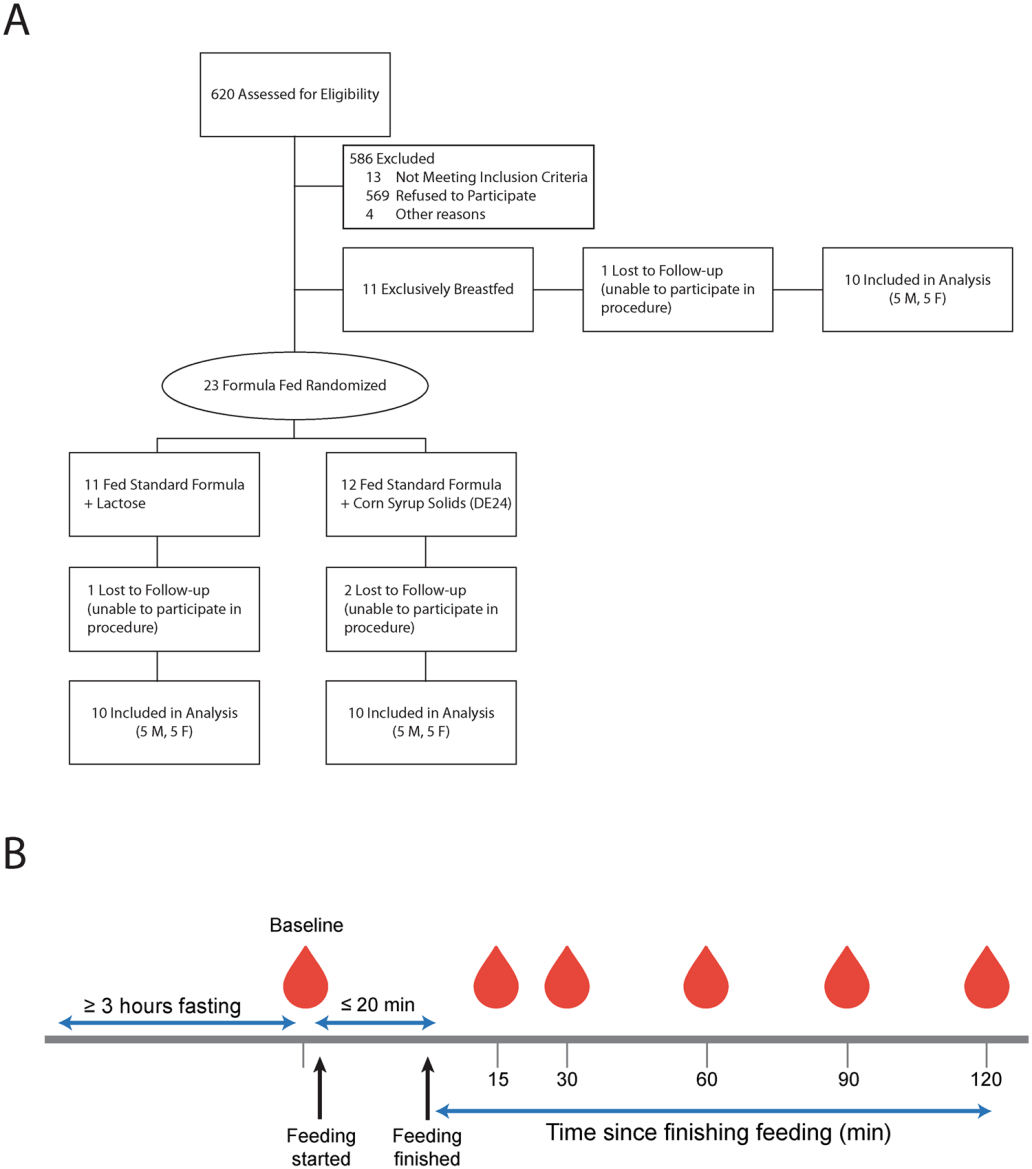
Study design. (**A**) Flow diagram of participant recruitment. (**B**) Study design.

Between June 2013 and December 2014, study staff screened charts from the delivery ward at Umeå University hospital, Umeå, Sweden. Inclusion criteria were healthy term infants (37–42 weeks gestation) with birth weight 2500–4500 g. Exclusion criteria were maternal diabetes, gestational diabetes, or any other complication during pregnancy and delivery, and any medical condition affecting the infant. Families with infants at 2 months of age were contacted and informed about the study. Of 620 infants screened for eligibility, 586 were not included. The main reason was that the family was not interested in participating in the study (N = 569). Thirteen infants did not meet inclusion criteria and 4 infants were excluded due to other reasons (difficulties in understanding Swedish/the study procedure). In all, 23 formula-fed infants and 11 breast-fed infants were included at 3 months (+/−2 weeks) of age. Formula-fed infants were further randomly assigned to receive either CSS- or lactose-based infant formula using a computer-generated randomization scheme stratified for sex (Fig. 4A).

Parents were provided with experimental formulas to acclimatize their infants to the taste prior to the test day (1–3 feedings). Experimental formulas consisted of carbohydrate-free infant formula (SHS Carbohydrate-Free Mixture, Nutricia Nordica AB, Solna, Sweden) as base and either a sachet of 6.54 g/100 g of lactose (Leprino Foods, Lemoore West, CA) or 6.54 g/100 g of CSS (DE24) (Roquette Freres, Lestrem, France). Sachets containing carbohydrate (lactose or CSS) were identical in appearance and marked with a letter. Study staff and parents were blinded to the intervention. Experimental formulas were prepared by dissolving 14 g of lactose or CSS in 200 mL of water, followed by mixing with the same amount (weight) of carbohydrate free mixture. The macronutrient composition of human milk, CSS- and lactose-based formulas are presented in Supplementary Table 3.

On the experimental day, subjects were admitted to the Pediatric Clinical Research Unit at Umeå University hospital in a semi-fasting state. Parents were instructed to fast their infants for at least 2 h, and numbing cream (Tapin®, Orifarm Generics AB, Odense, DK) was applied to a surface-exposed vein 1 h prior to insertion of an in-dwelling butterfly needle to minimize pain (Becton Dickinson, Plymouth, UK). In four infants an intra-venous line was not easily established and these infants were excluded (Fig. 4A). Fasting blood samples (1–1.5 mL) were drawn into microtubes with EDTA for plasma and without additives for serum (Becton Dickinson). Parents were instructed to feed their infants (breastfeed, or feed lactose- or CSS-based formula) for ~20 min. A research assistant prepared the formula, and a study nurse supervised the feeding. The amount consumed was calculated by subtracting the weight of the infant before from the weight after feeding for breast-fed infants, or measuring the amount left in the bottle for formula-fed infants. After feeding was finished, blood samples (1–1.5 mL) were drawn at 15, 30, 60, 90 and 120 min (Fig. 4B). Plasma (immediately) and serum (after 50 min at room temperature) were separated by centrifugation at 3,000 rpm for 10 min at room temperature. Plasma and serum were aliquoted, immediately frozen at −20 °C, and stored at −70 °C until further analysis.

### Ethical Considerations

Approval of the trial was obtained from the local ethical review board in Umeå (2013-152-31 M) and registered at www.clinicaltrails.gov (identifier NCT02441296) April 8, 2015. This study was conducted in accordance with the Declaration of Helsinki and Good Clinical Practice guidelines. Parents provided written informed consent for their infants.

### Serum insulin analysis

Serum insulin was analysed using a sandwich ELISA technique according to the manufacturer’s protocol (Merck Millipore Corporation, Darmstadt, Germany). Absorbance at 370 nm was measured on a SpectraMax 340 Microplate Reader (Molecular Devices Sunnyvale, CA, USA) and concentration was calculated as the mean value of duplicate analyses against a standard with SoftMax Pro 2.6.1 software using a 4-parameter logistic equation. Samples with % coefficient of variation (%CV) >10 were re-analysed. Two kit controls were analysed for intra assay variation to 3.4%. Using a serum control, the inter- and intra-assay variation was 9.5% and 3.0%, respectively, calculated as the mean of duplicate determinations in five separate assays. Concentrations are reported in μIU/mL.

### Non-esterified fatty acid (NEFA) analysis

NEFA analysis was performed immediately after plasma sampling to avoid lipolysis using an enzymatic colorimetric method (NEFA, Wako Chemicals GmbH, Neuss, Germany). Concentrations are reported in μmoles/L.

### NMR-based metabolome profiling

EDTA plasma samples were removed from −70 °C storage and allowed to thaw, after which they were filtered through a 3,000 MW cutoff filter (Amicon, 0.5 mL capacity, Millipore, Billerica, MA) to remove insoluble lipids and proteins. The volume of each filtrate was carefully measured and the appropriate amount of milliQ water was added to each filtrate to ensure a final volume of 199 μL. 8 μL of potassium phosphate buffer (1 M, pH 6.1) was added, followed by 23 μL of internal standard (5 mM 3-(trimethylsilyl)-1-propanesulfonic acid–d6 (DSS-d6)) dissolved in 0.2% NaN_3_ in 99.8% D_2_O. Sample pH was adjusted to 6.78 ± 0.05. NMR spectra were acquired on a Bruker Avance 600 MHz NMR spectrometer, and metabolites were quantified as previously described^13^ using Chenomx NMR Suite v8.1 (Chenomx Inc., Edmonton, Canada). Quantified metabolites included 2-hydroxybutyrate, 3-hydroxybutyrate, 3-hydroxyisobutyrate, acetate, acetoacetate, acetone, alanine, betaine, carnitine, choline, creatine, creatinine, dimethyl sulfone, ethanol, formate, glucose, glutamine, glycerol, glycine, isoleucine, lactate, leucine, lysine, methanol, methionine, N, N-dimethylglycine, o-acetylcarnitine, ornithine, proline, pyruvate, tyreonine, tyrosine, urea, valine, and *myo*-inositol. Concentrations are reported in μmoles/L.

### Statistical analyses

This was a pilot study, and the primary outcome was glucose concentration. Other metabolites were assessed as secondary outcomes. Each metabolite concentration was log_10_ transformed to reduce data heteroscedasticity. Principal component analysis (PCA) was performed on the log_10_-scaled data as an unsupervised clustering approach to identify similarities or differences between groups. Data were mean centered, and the score plot and variable correlations were visualized using ggplot2 in R.

To determine the grouping difference over time, two-way repeated measures ANCOVA (rmANCOVA) based on a linear mixed model was performed using nlme package in R for each log_10_ scaled metabolite concentration, where baseline measurements were used as covariates.

To evaluate the group difference in anthropometric data as well as differences at specific time points in plasma metabolites and NEFA, as well as serum insulin concentrations, one-way ANOVA followed by post-hoc HSD Tukey multiple comparison tests were performed. Data are presented as mean ± SD. P-values < 0.05 were considered statistically significant.

Area under the curve (AUC) analysis was computed using the trapezoid method as part of the pracma package in R based on serum insulin and plasma glucose concentrations with baseline adjustment (each post-meal measurement divided by the corresponding baseline reading) or without. Linear regression analyses were performed to determine the association between serum insulin and plasma glucose, leucine, isoleucine or valine, as well as plasma NEFA and ketone concentrations and visualized using ggplot2.

## Electronic supplementary material

Supplementary Material
