# Supplementary material for: Postprandial metabolic response of breast-fed infants and infants fed lactose-free vs regular infant formula: A randomized controlled trial
Source: Sci Rep. 2017 Jun 16;7:3640. doi: 10.1038/s41598-017-03975-4 (PMC5473881; doi:10.1038/s41598-017-03975-4)
Supplement: Supplementary file 1 — Supplementary Material [file 41598_2017_3975_MOESM1_ESM.pdf]

Supplementary Material:

Postprandial metabolic response of breast-fed infants and infants fed lactose-free vs regular infant formula. A randomized controlled trial.

Carolyn M. Slupsky[1], PhD; Xuan He[1], BSc; Olle Hernell[2], MD, PhD; Yvonne Andersson[2], RN; Colin Rudolph[3], MD, PhD; Bo Lönnerdal[1], PhD; Christina West[2], MD, PhD.

[1]Department of Nutrition, University of California, Davis, USA; [2]Department of Clinical Sciences/ Pediatrics, Umeå University, Sweden; [3]Mead Johnson Nutrition.

Contains: Supplementary Table 1, Table 2, and Table 3, as well as Supplementary Figure 1, and Figure 2.

**Supplementary Table 1.** Characteristics of infants enrolled in this study.				
	**Formula-fed (CSS-based)**	**Formula-fed (lactose-based)**	**Breastfed**	**p value**
**Mothers age at delivery** (y)	31.0 (26.4-34.1)	28.7 (25.8-32.0)	31.6 (26.6-34.8)	ns
**Parity**	1.0 (1.0-2.0))	1.5 (1.0-2.0)	2.0 (1.0-2.3)	ns
**University education (%)**				
Mother	70	40	80	ns
Father	50	10	50	ns
**Delivery mode**				ns
Vaginal	8	10	9	
Caesarean section	2	0	1	
**Gestational age**				
Weeks	39.7 (39.2-40.7)	40.9 (39.4-41.2)	40.4 (40.0-41.0)	ns
**Length**				
At birth (cm)	50.0 (49.0-50.5)	51.0 (48.8-51.3)	51.0 (50,0-51.0)	ns
At day of test meal (cm)	59.2 (58.6-61.0)	60.6 (58.4-61.3)	60.8 (59.4-61.8)	ns
**Weight**				
At birth (g)	3510 (3240.3665)	3500 (3291-3641)	3663 (3384-4076)	ns
At day of test meal (g)	6270 (5870-6440)	6090 (5730 (6650)	6240 (5560-6660)	ns
**Head circumference**				
At birth (cm)	35.0 (34.8-35.5)	34.8 (34.0-36.1)	35.5 (34.5-36.6)	ns
At day of test meal (cm)	40.3 (40.0-41.2)	40.2 (39.4-41.6)	41.2 (40.9-41.4)	ns
**Age at test meal** (days)	86.5 (83-88)	85 (81.5-89)	88 (85-89.5)	ns

Supplementary Table 2. Metabolites significantly different after feeding.

Metabolite	Breastfed vs MDX-based formula-fed	Breastfed vs. lactose-based formula-fed
Metabolites that were significantly different in both semi-fasting state (Table 1) and postprandial response:		
**Acetate**	**(p = 0.13)**	√
**Acetoacetate**	√	
**Acetone**	√	√
**Formate**	√	√
**Methanol**	√	√
***myo*-inositol**	**(p = 0.06)**	√
**2-hydroxybutyrate**	√	√
**3-hydroxyisobutyrate**	√	√
**Choline**	√	√
**Creatine**	√	√
**Dimethyl sulfone**	√	√
**Proline**	√	√
**Threonine**	√	√
**Urea**	√	√
**Valine**	√	√
Metabolites that were only significant in the postprandial response:		
**Betaine**		√
**Alanine**	√	√
**Isoleucine**	√	√
**Lactate**	√	
**Leucine**	√	√
**Lysine**	√	√
**Methionine**	√	√
**Tyrosine**	√	√

Statistical significance was determined based on repeated measures ANCOVA. The p-values from the multiple comparisons were adjusted by FDR. √**:** $p<0.05$. *myo*-Inositol was significant before but not after FDR adjustment. Higher in the breast-fed group: green; higher in the MDX-based formula-fed group: blue; higher in the lactose-based formula-fed group: red.

Supplementary Table 3. Macronutrient composition of CSS- and lactose-based infant formulas as well as human milk.

	**Lactose- based formula**	**CSS- based formula**	**Human milk ***
**Energy (kcal/100mL)**	**73.16 ****	**73.16****	**65 - 70**
**Protein (g/100mL)**	**2**	**2**	**0.9-1.2**
**Fat (g/100mL)**	**4.24**	**4.24**	**3.2-3.6**
**Lactose (g/100mL)**	**6.54**	**<0.01**	**6.7-7.8**
**Glucose (g/100mL)**	**<0.01**	**6.54**	**0.027**

* The values representing human mature/term milk are taken from[1]. Glucose concentration was estimated from the average glucose concentration as presented in[2].

** Energy for the lactose and maltodextrin-based formulas was estimated from the energy of the carbohydrate-free formula (47 kcal/100 mL) + the energy from the added carbohydrate.

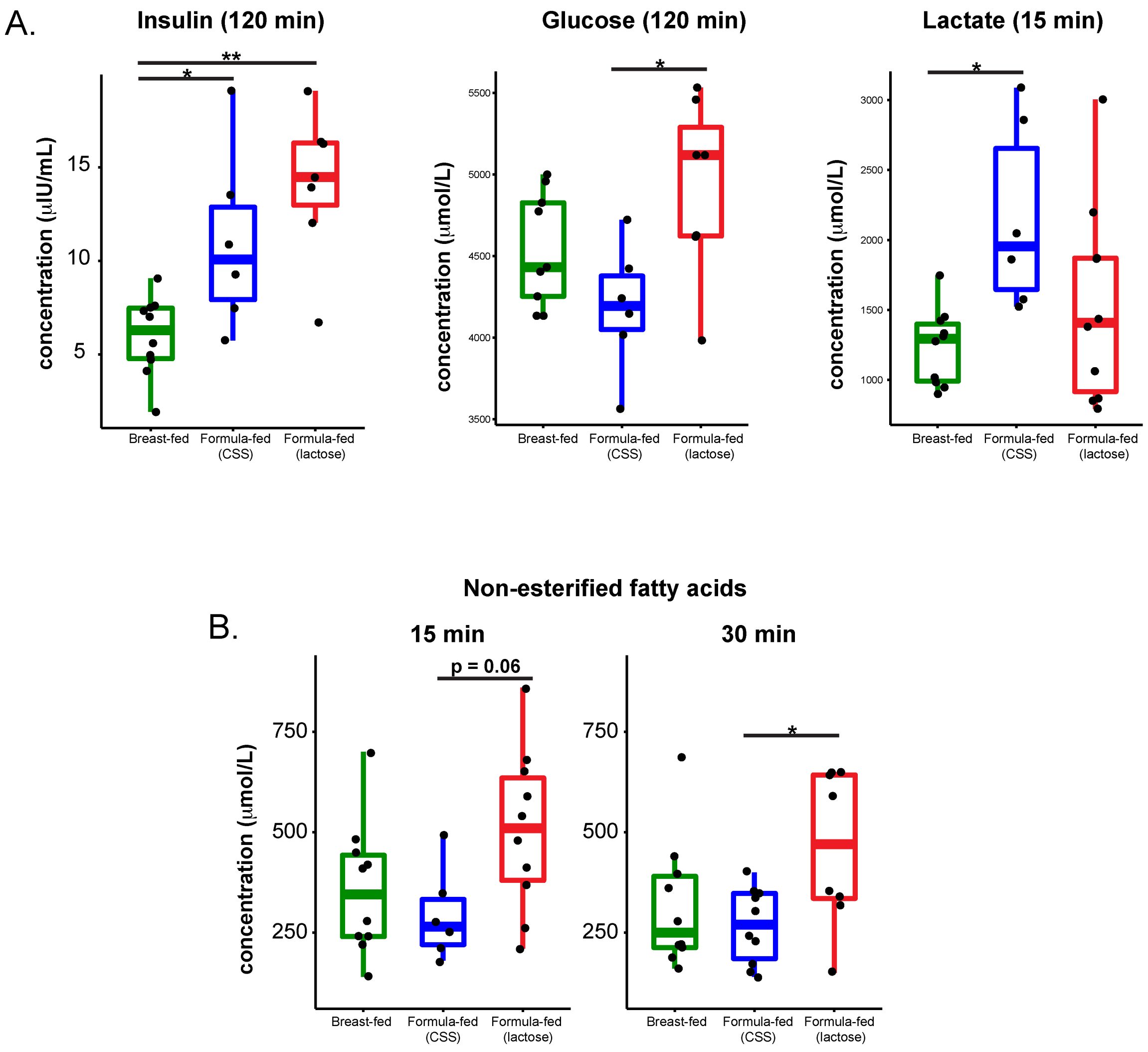

Supplementary Figure 1: Postprandial insulin, glucose, lactate and NEFA concentrations. (A) Insulin, and glucose at 120 min, and lactate at 15 min after feeding. (B) NEFA concentrations at 15 and 30 min after feeding. In green are breastfed infants, blue are CSS (DE24) formula-based infants, and red are lactose formula-based infants. * $p < 0.05$; ** $p < 0.0005$. P-values were computed based on one-way ANOVA followed by post-hoc Tukey HSD test.

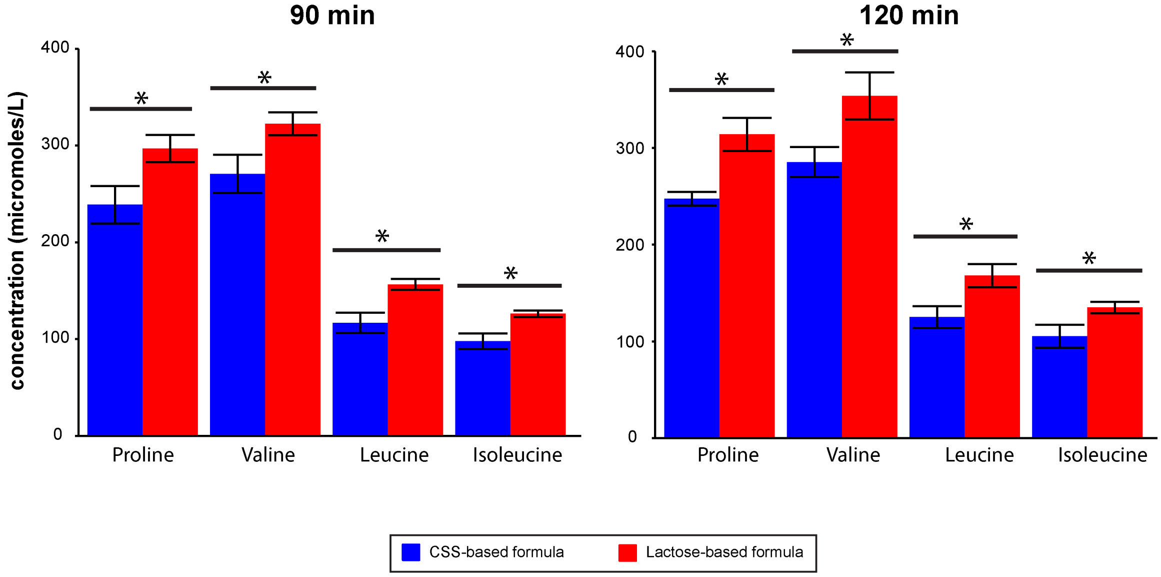

Supplementary Figure 2: CSS-based and lactose-based formulas elicit different post-prandial amino acid profiles at 90 and 120 min. Isoleucine, leucine, valine, and proline were significantly different between infants fed CSS-based or lactose-based formulas at 90 and 120 min. * $p<0.05$ by t-test.

REFERENCES:

1. Ballard O, Morrow AL. Human milk composition: Nutrients and bioactive factors. *Pediatr Clin North Am*. 2013;60(1):49-74.

2. Smilowitz JT, O'Sullivan A, Barile D, German JB, Lönnerdal B, Slupsky CM. The human milk metabolome reveals diverse oligosaccharide profiles. *J Nutr*. 2013;143(11):1709-1718.
